# Therapeutic Effect and Safety of Granulocyte Colony-Stimulating Factor Therapy for Acute-On-Chronic Liver Failure: A Systematic Review and Meta-Analysis of Randomized Controlled Trials

**DOI:** 10.3389/fmed.2021.784240

**Published:** 2021-11-16

**Authors:** Xiaoxue Hou, Yuwen Li, Hui Yuan, Jinyuan Cai, Rui Liu, Jun Li, Chuanlong Zhu

**Affiliations:** ^1^Department of Infectious Disease, The First Affiliated Hospital of Nanjing Medical University, Nanjing, China; ^2^Department of Pediatrics, The First Affiliated Hospital of Nanjing Medical University, Nanjing, China; ^3^Department of Tropical Diseases, The Second Affiliated Hospital of Hainan Medical University, Haikou, China; ^4^Laboratory of Infectious Disease, The First Affiliated Hospital of Nanjing Medical University, Nanjing, China

**Keywords:** granulocyte colony-stimulating factor, acute-on-chronic liver failure, end stage liver disease, hepatic insufficiency, randomized controlled trial

## Abstract

**Background and Aims:** Granulocyte colony-stimulating factor (G-CSF) has been proposed as a therapeutic option for patients with acute-on-chronic liver failure (ACLF). However, its clinical efficacy remains debatable. This study aimed to synthesize available evidence on the efficacy of G-CSF in ALCF.

**Methods:** The Cochrane Library, CNKI, MEDLINE, EMBASE, Cochrane Central Register of Controlled Trials (CENTRAL), and ClinicalTrials.gov were searched from inception until September 2021. After qualitative evaluation of the included literature, the included studies were analyzed.

**Results:** Seven studies were included in this meta-analysis. Overall, G-CSF therapy was not associated with a reduced risk of death (30-day survival, OR = 1.55, 95% CI: 1.00, 2.38, *P* = 0.05; 60-day survival, OR = 1.50, 95% CI: 0.95, 2.36, *P* = 0.08; 90-day survival, OR = 1.61, 95% CI: 0.99, 2.62, *P* = 0.05) or complication including occurrence of infections infection (OR = 0.66, 95% CI: 0.41, 1.05, *P* = 0.08), bleeding (OR = 1.50, 95% CI: 0.58, 3.89, *P* = 0.41), and hepatorenal syndrome (OR = 0.56, 95% CI: 0.25, 1.24, *P* = 0.15). Moreover, it had no obvious beneficial effects on the model of end-stage liver disease score (30-day SMD = −3.31, 95%CI: −7.42, 0.81, *P* = 0.12; 60-day SMD = −1.23, 95% CI: −5.21, 2.75, *P* = 0.54; 90-day SMD = −2.29, 95%CI: −4.94, 0.37, *P* = 0.09). Sensitivity analyses showed that patients in Asia had improved survival (30-day OR = 2.76, 95%CI: 1.43, 5.35, *P* = 0.003; 60-day OR = 2.83, 95% CI: 1.39, 5.73, *P* = 0.004; 90-day OR = 2.92, 95% CI: 1.34, 6.36, *P* = 0.007).

**Conclusions:** Our findings suggest that, currently, G-CSF cannot be recommended for the treatment of ACLF.

## Introduction

Acute-on-chronic liver failure (ACLF) is a kind of short-term liver damage due to acute causes of chronic liver disease; it is accompanied by the failure of one or more extrahepatic organs, and the short-term risk of death is high (1). Currently, there is no specific treatment for ACLF, and liver transplantation is the definitive treatment for ACLF. However, many patients cannot benefit from liver transplantation because of limited organ availability, high cost, transplant-related complications, and lifetime immunity-related side effects (2, 3). Therefore, alternative treatment strategies for liver transplantation are being sought.

Granulocyte colony-stimulating factor (G-CSF) is a glycoprotein that can mobilize immune cells, stimulate bone marrow and stem cell production, and it has immune regulation and regeneration capabilities. Previous studies have been reported encouraging results on the use of G-CSF in animal models. G-CSF is found to mobilize hematopoietic stem cells, induce liver regeneration, and improve survival. However, Engelmann et al. (4) found that G-CSF increased Toll-like receptor-mediated inflammation, which led to an increase in mortality. In human studies, a few small randomized clinical trials have demonstrated not only improvement in liver function with G-CSF but also significant survival benefit compared with standard medical therapy for ACLF (5–7). On the contrary, other clinical trials reported that the use of G-CSF in ACLF patients did not result in survival benefits (4, 8), which has caused widespread concern. Therefore, in this study, we conducted a meta-analysis of randomized controlled trials (RCTs) to compare the risk of death and infection between ACLF patients treated with G-CSF and ACLF patients who did not receive G-CSF.

## Methods

### Literature Search

**Figure 1:**
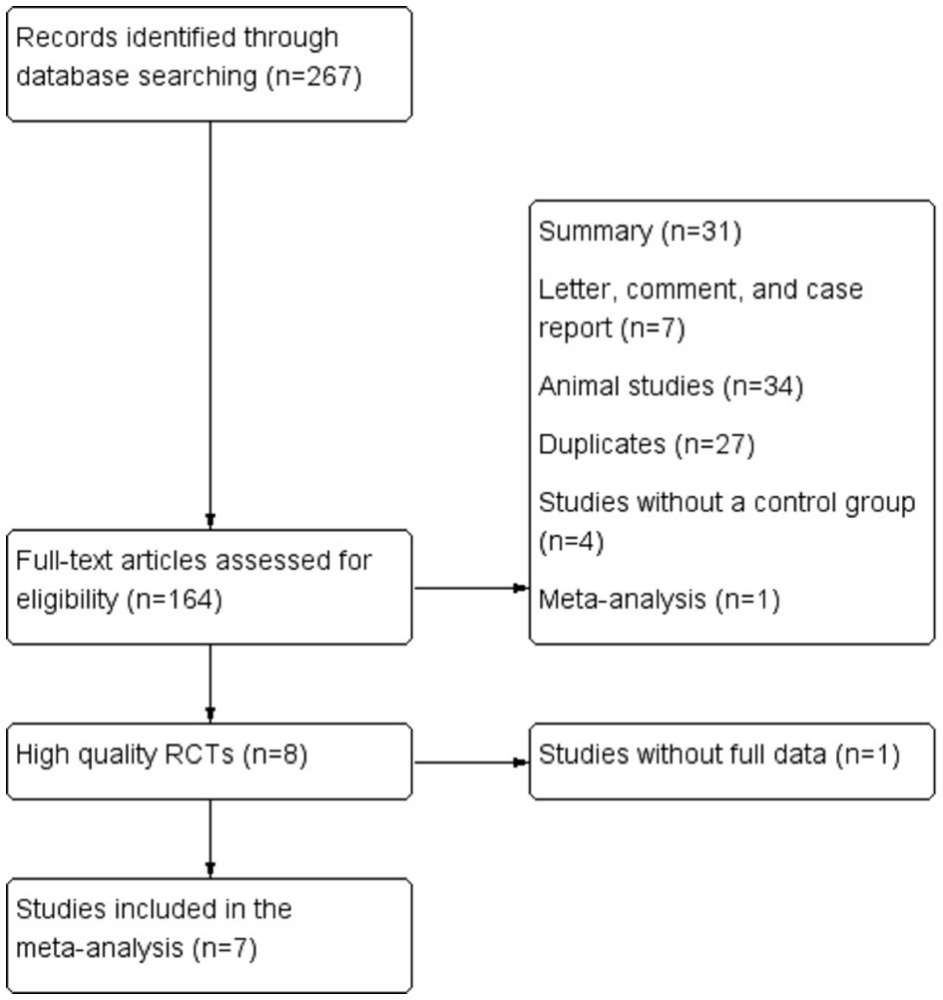
Flow chart of the selection of studies for inclusion in the meta-analysis. RCT, randomized controlled trial.

We searched for RCTs involving ACLF patients treated with G-CSF from electronic medical databases, including the Cochrane Library, CNKI, MEDLINE, EMBASE, Cochrane Central Register of Controlled Trials (CENTRAL), and ClinicalTrials.gov, from inception to September 14, 2021. Key search terms were “granulocyte colony-stimulating factor,” “acute-on-chronic liver failure,” “hepatic insufficiency,” “end stage liver disease,” and “randomized controlled trial.” MeSH terms and free-text terms, as well as variations of root words, were combined within each database. No language restrictions were applied during the search. The reference lists of eligible articles and relevant review articles were also checked to identify additional studies. The detailed search strategies are outlined in Figure 1.

### Study Inclusion and Exclusion Criteria

The inclusion criteria were as follows: (1) RCTs, (2) patients diagnosed with ACLF, (3) patients in the experimental group received G-CSF therapy, and patients in the control group received conventional treatment, and (4) availability of clinical outcomes. The primary outcomes were short-term survival rate. The secondary outcomes included the scores of Model for End-Stage Liver Disease (MELD), risk of bleeding, occurrence of infections and hepatorenal syndrome. The exclusion criteria were (1) animal-based review articles or case reports, and (2) research in which valid data could not be extracted from the full text.

### Data Extraction and Quality Score

Data extraction was performed independently by two authors using standardized data collection forms, and disagreements were resolved through discussion with another author. The following details were extracted from the included studies using a predefined data form: study first author, year of publication, country, and the number of participants in the experimental and control groups, the specific etiology of liver failure, the dose, administration, and duration of G-CSF treatment. Each trial was assessed using the Cochrane risk of bias tool. The standard criteria included the following domains: random sequence generation, allocation concealment, blinding of participants and personnel, blinding of outcome assessment, incomplete outcome data, selective reporting and other bias.

### Statistical Analysis

Review Manager (version 5.4) software was used for the data merging and processing. For categorical variables in the study, the standardized mean difference (SMD) and odds ratio (OR) was used as the effect index to calculate the combined value and 95% confidence interval (CI). The Mantel-Haenszel test was used to test the heterogeneity of the included studies. If *I*^2^ ≤ 50% (*P* ≥ 0.05), indicating that the differences in the studies were not statistically significant, the fixed-effects model was used for analysis. If *I*^2^ > 50% (*P* < 0.05), indicating that there was significant heterogeneity, the random-effects model was used. Obvious clinical heterogeneity was evaluated by removing a single study and repeating the meta-analysis. Statistical significance was set as *P* < 0.05. Publication bias was evaluated with a funnel plot.

## Results

### Selection of Eligible Studies

Following the search strategy described in Figure 1, 267 studies were initially identified based on the assessment of the titles and abstracts. We excluded 260 studies considering the predefined criteria. Seven eligible studies were finally included in the meta-analysis (5, 6, 8–12).

### Characteristics of the Included Studies

Seven studies involving 479 ACLF patients included G-CSF treatment (*n* = 240) and control (*n* = 239) groups. The baseline characteristics, including the study design, treatment methods for each group, sample sizes of each group (Table 1).

**Table 1:** Basic characteristics of included literature.

**Authors**	**Country**	**Etiology**	**Sample size (Exp/Con)**	**Group of patients/ study design**	**G-CSF doses**	**Age (years, range)**	**Sex ratio (No. of males, %)**	**MELD score at baseline**	**CTP score at baseline**	**Number of people surviving 30 days**	**Number of people surviving 60 days**	**Number of people surviving 90 days**	**Number of infections**	**Number of HRS**	**Number of bleeding**
Garg et al. (5)	India, Asia	AH and HBV	23	G-CSF	5 μg/kg/day (5 days) and every 3 days (1 month); subcutaneously	40 (30–65)^*^	20 (87%)	29 (21–40)^*^	12 (11–14)^*^	–	16	–	3	–	2
			24	Placebo+ SMT		40 (19–55)^*^	21 (87%)	31.5 (20–40)^*^	12 (10–14)^*^	–	7	–	10	–	2
Duan et al. (6)	China, Asia	HBV	27	G-CSF+SMT	5μg/kg/days; 6 days; subcutaneously	43.5(29–63)^*^	22 (81.5%)	25.11 ± 3.30^**^	12.17 ± 1.47^**^	–	–	13	3	2	5
			28	SMT		45.9(22–65)^*^	22 (78.6%)	26.30 ± 4.12^**^	12.25 ± 1.29^**^	–	–	6	7	6	3
Xiang et al. (11)	China, Asia	HBV	49	G-CSF+SMT	300 μg/days subcutaneously	41.72 ± 10.11^**^	40 (83.3%)	23.78 ± 3.68^**^	–	45	–	–	12	4	–
			50	SMT		45.62 ± 10.36^**^	42 (84%)	24.62 ± 4.45^**^	–	32	–	–	16	4	–
Saha et al. (9)	India, Asia	HBV	16	G-CSF+SMT	5 μg/kg/day (5 days); subcutaneously	48 (22–62)^*^	16(100%)	24.5 (21–32)^*^	12 (10–14)^*^	–	14	14	–	1	0
			16	SMT		39 (18–55)^*^	12(75%)	25.5 (21–35)^*^	12 (10–13)^*^	–	13	8	–	3	2
Sharma et al. (10)	India, Asia	HEV and HAV	15	G-CSF+SMT	5 mcg/kg/day (5 days); subcutaneously	7.53 ± 3.7^**^	7(46.6%)	–	12 ± 1.4^**^	10	8	–	–	–	–
			16	SMT		6.31 ± 4.9^**^	12(75%)	–	12.75 ± 0.85^**^	6	6	–	–	–	–
Haque et al. (12)	Japan, Asia	HBV	22	G-CSF+EPO	5 μg/kg/day(6 days); subcutaneously	42.64 ± 10.39^**^	19(86.4%)	27.64 ± 4.6^**^	–	14	11	8	1	5	4
			17	SMT		42.18 ± 13.06^**^	12(70.6%)	29.47 ± 5.5^**^	–	10	5	5	3	5	0
Engelmann et al. (8)	Multicentric, Europe	AH	88	G-CSF+SMT	5 μg/kg/day (5 days) and every 3 days (1 month); subcutaneously	54.4 ± 10.2^**^	50 (56.8%)	24.4 ± 6.3^**^	–	42	29	27	71	–	–
			88	SMT		57.1 ± 9.6^**^	61 (69.3%)	24.5 ± 6.1^**^	–	43	31	26	69	–	–

*Con, control group; Exp, experimental group; G-CSF, granulocyte colony-stimulating factor; SMT, standard treatment; EPO, erythropoietin; AH, Alcoholic hepatitis; HBV, Hepatitis B virus; HEV, hepatitis E virus; AVH, acute viral hepatitis*.

* *Expressed as median*.

** *Expressed as mean ± SD*.

### Quality Assessment of the Included Studies

**Figure 2:**
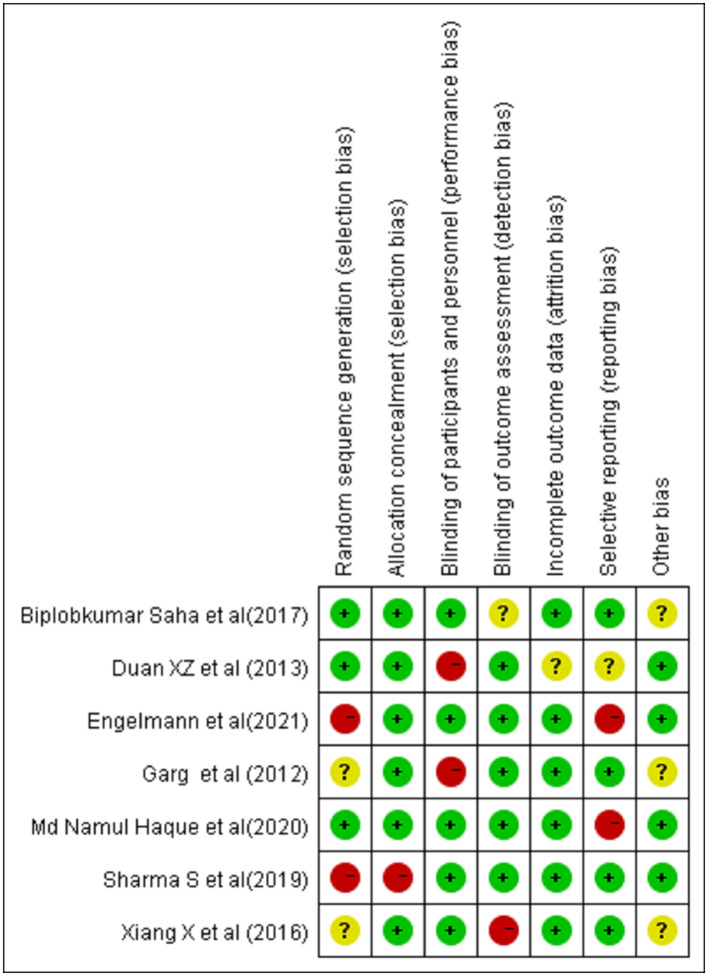
Assessment of risk of bias.

The details of the risk of bias tool are shown in Figure 2.

### Survival Rate

**Figure 3:**
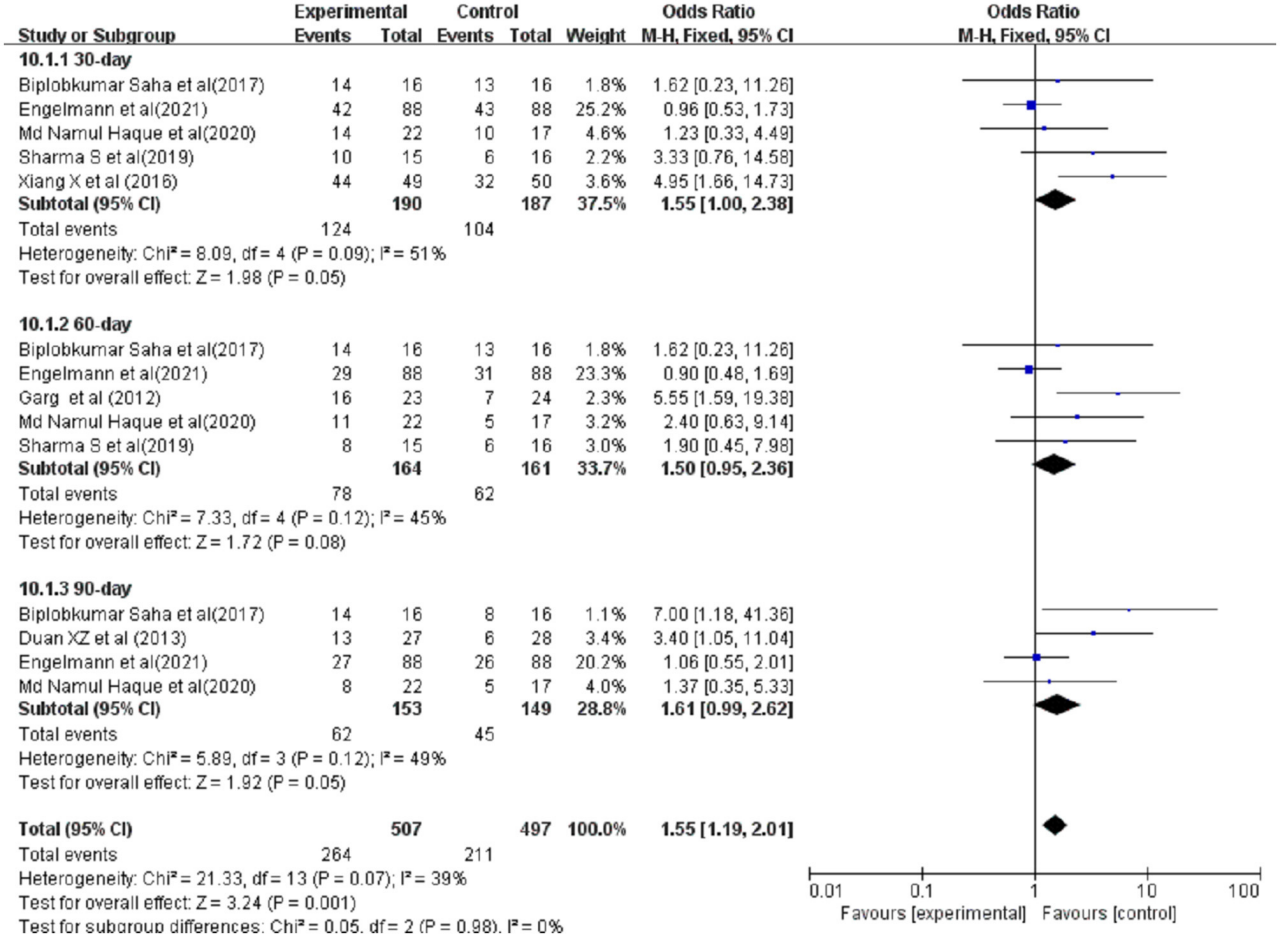
Survival rate between patients with ACLF. Treated by G-CSF and controls.

Seven studies were included in the analysis of survival rate, compared with conventional treatment, G-CSF therapy was not associated with a reduced risk of death (30-day survival, OR = 1.55, 95% CI: 1.00, 2.38, *P* = 0.05; 60-day survival, OR = 1.50, 95% CI: 0.95, 2.36, *P* = 0.08; 90-day survival, OR = 1.61, 95% CI: 0.99, 2.62, *P* = 0.05) (Figure 3).

### Clinical Severity Indices

**Figure 4:**
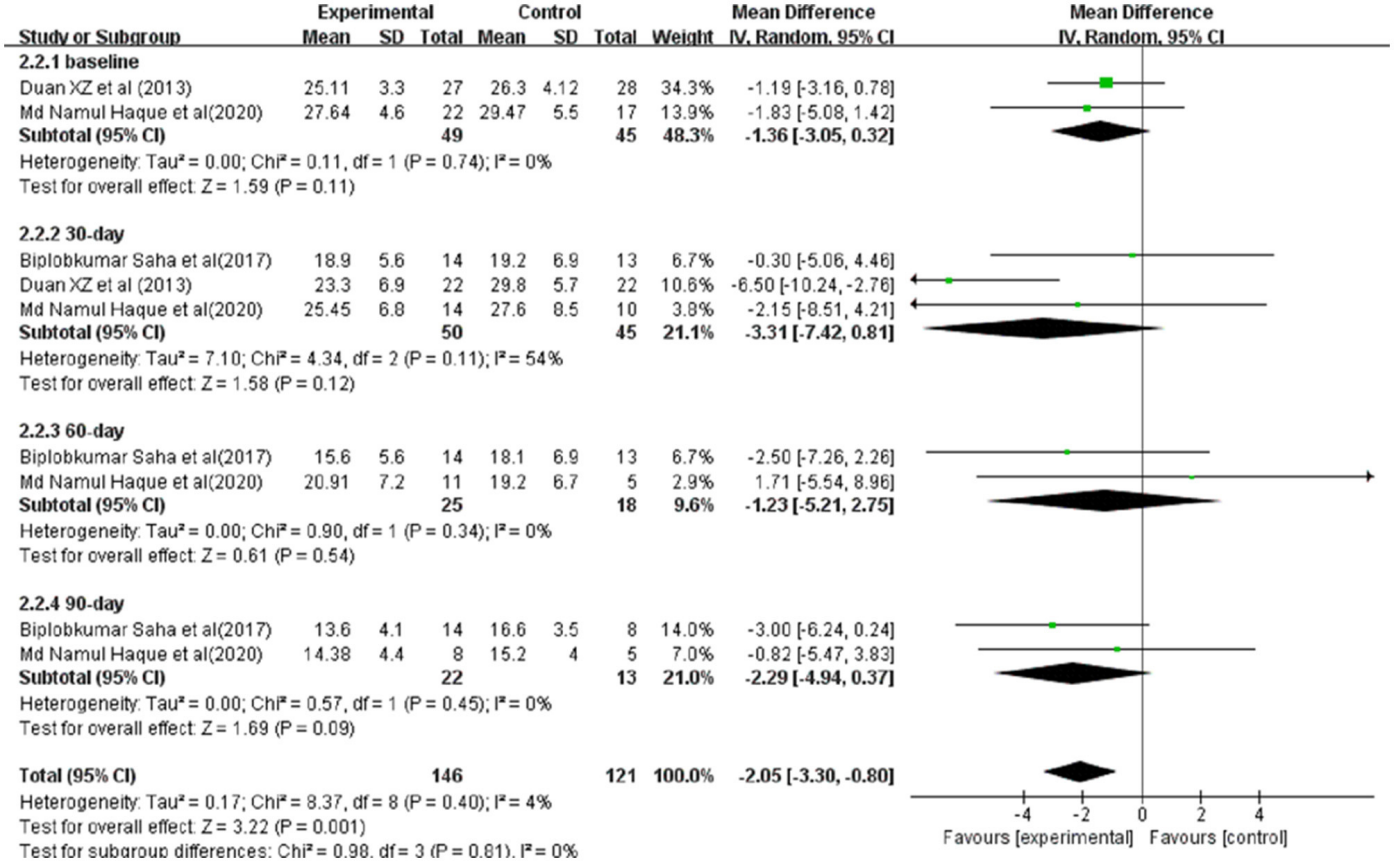
Improvement in clinical severity indices between patients with ACLF. Treated by G-CSF and controls.

The scores of Model for End-Stage Liver Disease (MELD) in ACLF patients for meta-analysis were available from three studies (Figure 4). Before and after treatment, no significant difference was observed between the experimental and control groups (Before treatment: SMD = −1.36, 95% CI: −3.05, 0.32, *P* = 0.11; After treatment: 30-day SMD = −3.31, 95%CI: −7.42, 0.81, *P* = 0.12; 60-day SMD = −1.23, 95% CI: −5.21,2.75, *P* = 0.54;90-day SMD = −2.29, 95%CI: −4.94,0.37, *P* = 0.09).

### Occurrence of Infections

**Figure 5:**
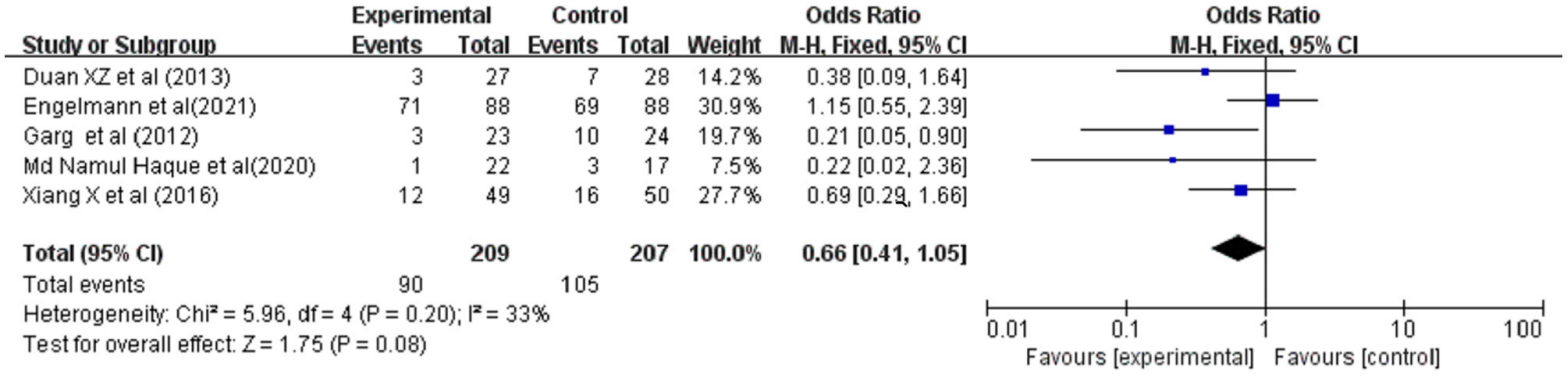
Pooled estimate rate for infection between patients with ACLF. Treated by G-CSF and controls.

Secondary infections were reported in 5 studies involving 416 ACLF patients, 209 were in the G-CSF treatment group and 207 were in the control group. Owing to the low heterogeneity (*I*^2^ = 33%), a fixed-effects model was adopted. The meta-analysis showed that patients receiving G-CSF did not have a significantly reduced risk of infections compared with traditional treatment (OR = 0.66, 95% CI: 0.41, 1.05, *P* = 0.08) (Figure 5).

### Occurrence of Bleeding

**Figure 6:**
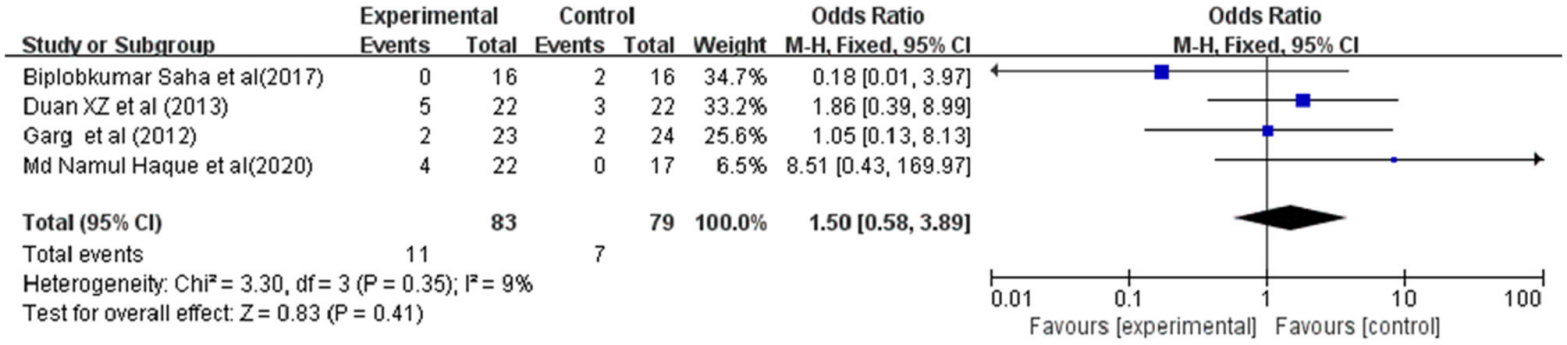
Pooled estimate rate for bleeding between patients with ACLF. Treated by G-CSF and controls.

Bleeding was reported in 4 studies involving 162 ACLF patients, 83 were in the G-CSF treatment group and 79 were in the control group. Owing to the low heterogeneity (*I*^2^ = 9%), a fixed-effects model was adopted. The meta-analysis showed that compared with traditional treatment, patients receiving G-CSF did not have a significantly reduced risk of bleeding (OR = 1.50, 95% CI: 0.58, 3.89, *P* = 0.41) (Figure 6).

### Occurrence of Hepatorenal Syndrome

**Figure 7:**
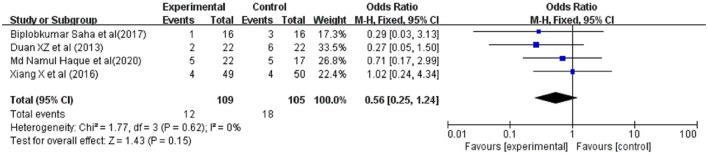
Pooled estimate rate for Hepatorenal syndrome between patients with ACLF. Treated by G-CSF and controls.

Hepatorenal syndrome (HRS) were reported in 4 studies involving 214 ACLF patients, 109 were in the G-CSF treatment group and 105 were in the control group. Owing to the low heterogeneity (*I*^2^ = 0%), a fixed-effects model was adopted. The meta-analysis showed that compared with traditional treatment, patients receiving G-CSF did not have a significantly reduced risk of HRS (OR = 0.56, 95% CI: 0.25, 1.24, *P* = 0.15) (Figure 7).

### Sensitivity Analyses

**Figure 8:**
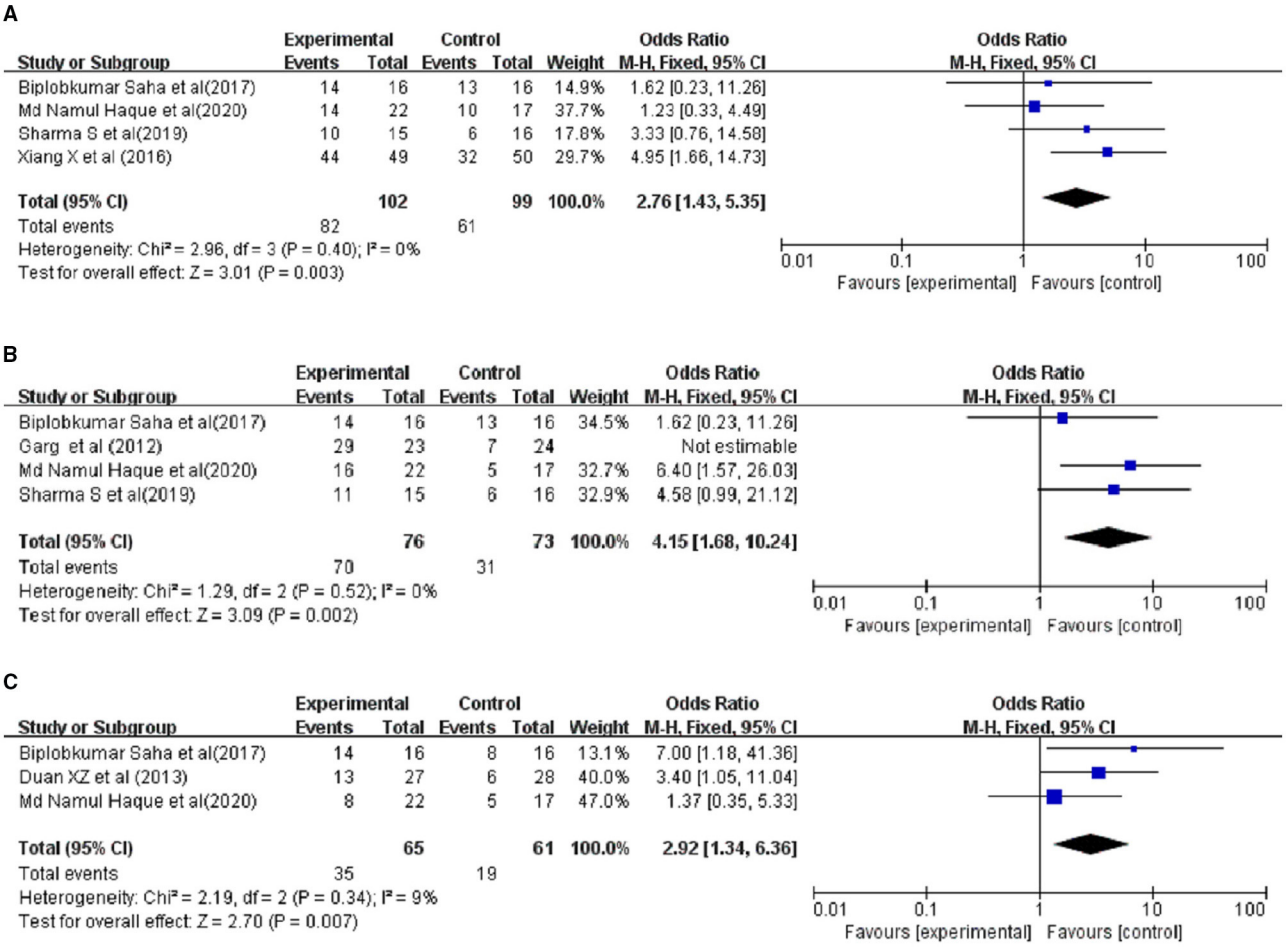
Sensitivity analysis. Treated by G-CSF and controls. **(A)** 30-day; **(B)** 60-day; **(C)** 90-day.

The sensitivity analysis showed that after excluding European studies, 4 Asian studies remained with low heterogeneity (30-day and 60-day *I*^2^ = 0%; 90-day *I*^2^ = 9%); considering these Asian studies, the patients' survival rate improved after the injection of G-CSF (30-day OR = 2.76, 95%CI: 1.43, 5.35, *P* = 0.003;60-day OR = 2.83, 95% CI: 1.39, 5.73, *P* = 0.004) (Figures 8A–C).

### Risk of Publication Bias

**Figure 9:**
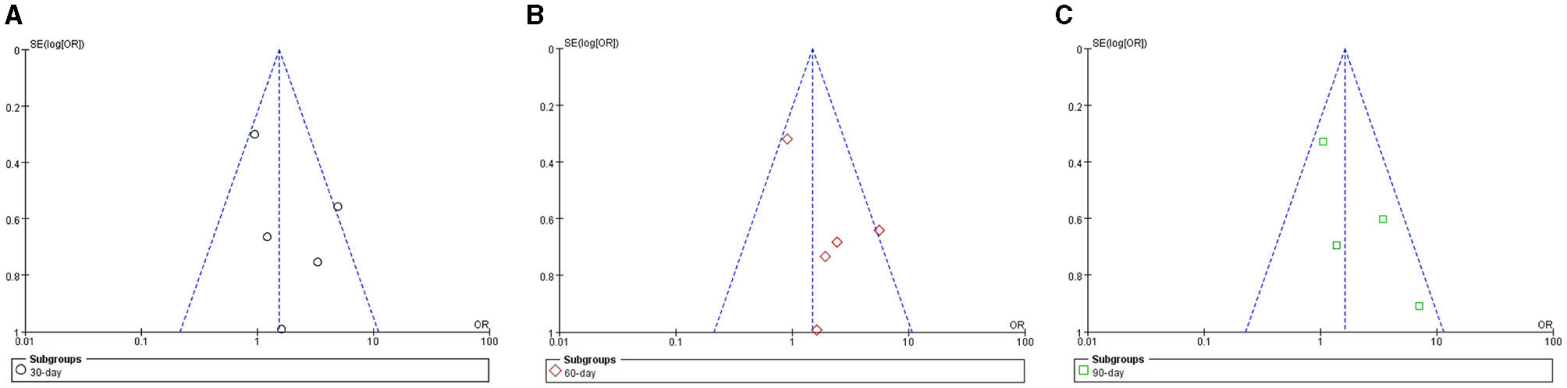
Funnel plot of meta-analyses for survival rate in ACLF patients. **(A)** 30-day; **(B)** 60-day; **(C)** 90-day.

Funnel plots of survival rate meta-analyses demonstrated asymmetry and suggested the presence of publication bias (Figures 9A–C).

## Discussion

ACLF is characterized by organ failure and high short-term mortality. Currently, there are no specific therapies for ACLF. Liver transplantation is the ultimate treatment for those who are acceptable candidates, but is limited by organ shortage and high frequency of contraindications in this group of patients (2, 3). Previous studies have shown that G-CSF can reduce the short-term mortality of ACFL patients (13). Yet the newly published clinical trial does not seem to support this conclusion (8). In this study, we conducted a comprehensive meta-analysis of 7 RCTs to evaluate the efficacy and safety of G-CSF in the treatment of ACLF patients. We failed to find a significant beneficial effect of G-CSF for patients with ACLF, unlike noted in a previous meta-analysis (14). Through sensitivity analysis of sources of heterogeneity, we found that etiologies of ACLF differ by region, with reactivation of hepatitis B more common in Asia, whereas alcoholic hepatitis are reportedly more common in European. We speculate the ultimate treatment outcome of patient may depend on the etiology of ACLF. In addition, different ACLF diagnostic criteria have led to considerable regional differences in ACLF recognition, onset and treatment timing, and final prognosis. In this meta-analysis, the admission criteria for ACLF patients in the Asian study were in accordance with APASL, and the admission criteria for patients in the European study were in accordance with the EASL-CLIF criteria. An intriguing and important finding of this study was significantly different disease progression among patients with ACLF at enrollment defined by APASL or EASL-CLIF Consortium. This may also affect the therapeutic effect of G-CSF on different ACLF patients.

Currently, it has been shown that G-CSF stimulates liver regeneration. G-CSF can stimulate bone marrow to release stem cells (CD34+), which could migrate to liver and differentiate into mature hepatocytes. It can also reduce the production of interferon-gamma, improve the local microenvironment of liver, promote liver repair, and improve liver injury. This translates into improved liver function, decreased risk of complications of liver disease, reduced risk of infections, and improved survival (15). The effect of G-CSF on liver regeneration may explain the survival benefit which was observed in Asian studies. The role of G-CSF in the latter stages of ACLF is limited due to the exhausted and destructed state of bone marrow ecology. In the Asian regional study included in this meta-analysis, G-CSF was generally used in the early stages of ACLF. In the included European RCTs trial, we found that ~70% of patients had cardiopulmonary failure and severe sepsis at the time of enrollment (8), which means they were in the end-stage of ACLF. Whether this condition affects our final results needs further exploration. In addition, studies have found that G-CSF requires a non-inflammatory environment to exert its protective effects on the liver. ACLF is characterized by increased white blood cell counts and plasma C-reactive protein levels. Patients often have a strong systemic inflammatory response (8, 16), and we speculate that G-CSF does not play a beneficial role in ACLF patients. Moreover, there were fewer studies in the European region in this study. Thus, more European clinical trials are needed to determine whether current results from the included European region trials were non-comprehensive. There were no clear conclusions concerning the usefulness of G-CSF in those with ACLF, although survival benefits were observed in Asian patients compared to European patients. The conflicting results between Asian and European studies led to a high degree of overall heterogeneity in the analysis, and it is unclear whether this difference can be explained by ethnic differences or patient selection. Based on our results, we do not recommend G-CSF as a definitive treatment for patients with ACLF.

The Model for End-Stage Liver Disease (MELD) has been established as a reliable indicator of short-term survival in patients with end-stage liver disease. In this meta-analysis, we comprehensively analyzed the clinical severity indices of ACLF. The results suggested that G-CSF therapy may not improve MELD scores, unlike what is noted in a previous meta-analysis (17). Also, patients with G-CSF therapy did not achieve significantly lower bleeding risk and the occurrence of HRS compared with standard medical treatment. ACLF has marked pathophysiological features, namely, susceptibility to infection. Moreover, bacterial infection is a major challenge for its treatment (18, 19). G-CSF is an immunomodulatory glycoprotein that exerts anti-inflammatory and immunomodulatory effects in the body, thereby reducing the occurrence of bacteremia and infection. This benefit of G-CSF may be particularly important in patients with ACLF. Nonetheless, in this meta-analysis, there was no significant difference in the risk of infection among patients receiving treatment. We found that in the study of Engelmann et al. almost 40% of patients had bacterial infection at enrollment (8). The role of G-CSF for ACLF patients with severe bacterial infection is debatable. G-CSF is helpful to prevent development of bacterial infection, but is not beneficial to treat it. It is important to clarify whether this affected the final results.

Several limitations of this meta-analysis should be mentioned. First of all, the high heterogeneity in some aggregate estimation results may have hindered the establishment of reliable conclusions and recommendations. Sensitivity analyses indicated that regions and different types of liver disease may be the main cause of heterogeneity. However, it is unclear whether other factors would lead to the results of the study, such as the degree of ACLF progress, differences between studies, and the dose and duration of G-CSF injections. Moreover, some data cannot be obtained from each study, resulting in limited strength of evidence for the results obtained. Secondly, the total sample size is small, which may affect the reliability of the analysis results to a certain extent, and has limited significance for clinical guidance. Lastly, different trials use different outcome parameters at different measurement time points to evaluate the treatment effect, making it difficult to use a limited statistical sample size at a specific time point to summarize reliable results.

No clear conclusion could be drawn regarding the usefulness of G-CSF in ACLF, although survival benefits were observed in Asian patients. The conflicting results between regions and different etiology of liver disease lead to a high degree of overall heterogeneity in the analysis. It is unclear whether these differences can be explained by ethnic differences or different liver failure causes. Moreover, different diagnostic criteria for ACLF caused different patient prognosis (20). We need more RCTs and high-quality literature are required to clarify the usefulness of G-CSF for ACLF treatment. In conclusion, based on our results, we do not recommend G-CSF as a definitive treatment for patients with ACLF.

## Data Availability Statement

The original contributions presented in the study are included in the article/supplementary material, further inquiries can be directed to the corresponding author.

## Author Contributions

XH, YL, and CZ conceived and designed the study. XH, HY, and JC selected the studies, collected the data, and drafted and revised the paper. XH, RL, and JL analyzed data. All authors interpreted the results, read, and approved the final version of the manuscript.

## Funding

This work was supported by the National Natural Science Foundation of China (Grant Nos. 81770591 and 81800778), Science and Technology Plan of Hainan Province (Clinical Research Center) (LCYX202103), and Hainan Province Clinical Medical Center.

## Conflict of Interest

The authors declare that the research was conducted in the absence of any commercial or financial relationships that could be construed as a potential conflict of interest.

## Publisher's Note

All claims expressed in this article are solely those of the authors and do not necessarily represent those of their affiliated organizations, or those of the publisher, the editors and the reviewers. Any product that may be evaluated in this article, or claim that may be made by its manufacturer, is not guaranteed or endorsed by the publisher.

## Abbreviations

ACLF: acute-on-chronic liver failure
CI: confidence interval
G-CSF: granulocyte colony-stimulating factor
OR: odds ratio
RCT: randomized controlled trial.
